# Quantification of plasma tau species containing the proline-rich region as a biomarker in Alzheimer’s disease

**DOI:** 10.1038/s41598-025-25864-x

**Published:** 2025-11-25

**Authors:** Mohammad Arastoo, Lewis K. Penny, Richard Lofthouse, Aaron Hitchcock, Anna Moelders, Sona Szalma, Andrew Porter, Soumya Palliyil, Charles R. Harrington, Claude M. Wischik

**Affiliations:** 1 Institute of Medical Sciences, University of Aberdeen, Aberdeen, UK; 2 Scottish Biologics Facility, University of Aberdeen, Aberdeen, UK; 3 GT Diagnostics (UK) Ltd., Aberdeen, UK; 4 TauRx Therapeutics Ltd., Aberdeen, UK

**Keywords:** Alzheimer’s disease, Tauopathy, Tau protein, Tau fragments, Polyclonal antibody, Biomarker, Biochemistry, Biomarkers, Neurology, Neuroscience

## Abstract

Tau-based blood biomarkers are increasingly recognised as important for the diagnosis of Alzheimer’s disease (AD). More than 60 proteolytic cleavage sites of tau have been identified, and current assays may miss critical information from some of the smaller protein fragments. By capturing a broader range of tau species, a polyclonal approach may offer greater interrogation of this complex “tauosome” and deliver valuable insights into the onset or progression of AD. A sheep was hyper-immunised with 2N4R tau113-251 peptide, encompassing the proline-rich region. An affinity-purified proline region polyclonal antibody (P.pAb) was derived from sheep serum, after four rounds of immunisation. Following characterisation of P.pAb, utility as a plasma biomarker/diagnostic agent for AD was assessed using a single molecular array (Simoa) assay in a selected cohort consisting of clinically diagnosed AD patients and age-matched cognitively unimpaired (CU) individuals. Two assays were considered for this assessment including pairing the P.pAb with itself (P.pAb-P.pAb) to capture and detect multiple tau fragments in plasma, and pairing pTau217 capture mAb with P.pAb (pTau217-P.pAb). The P.pAb showed high affinity towards full-length tau and 113–251 peptide immunogen and bound smaller 13-amino acid (aa) fragments throughout the proline rich region. The selected patient cohort was initially assessed by commercial neurofilament light (NfL) and pTau217 assays, the results of which were consistent with AD-related neurodegeneration in the AD sample and not in the CU group. The P.pAb-P.pAb and the pTau217-P.pAb assays were each able to distinguish between CU and AD groups; values were greater in AD (1.4-fold, *p* < 0.0001 and 2.8-fold, *p* < 0.001, respectively). By contrast, a commercial total-tau (T-tau) assay did not distinguish between the two groups. We demonstrate the feasibility of an immunodiagnostic approach based on the detection of tau species containing the proline-rich region. The development of an affinity-purified proline region-specific pAb, capable of detecting multiple tau species in plasma, provides the foundation for a novel approach with potential applications in AD diagnosis and monitoring of disease progression.

**Supplementary Information:**

The online version contains supplementary material available at 10.1038/s41598-025-25864-x.

## Introduction

Tau protein is intrinsic to the pathophysiology of Alzheimer’s disease (AD)^1,2^. A primary physiological function for tau within neurons is to bind to and stabilise microtubules^3,4^. Full-length tau protein consists of 4 distinct domains: an N-terminal acidic projection domain which extends away from the surface of the microtubule; a proline rich basic domain which is important for interaction with proteins that regulate its function; a microtubule binding domain (MTBR) that mediates microtubule assembly; and a short C-terminal region^5,6^.

In AD, pathologically aggregated tau spreads in a prion-like manner following a stereotypical pattern, originating in the locus coeruleus and transentorhinal cortex (Braak stage I–II), subsequently spreading to the limbic system (Braak stage III–IV) and the neocortex (Braak stage V–VI)^7^. Many post-translational modifications are involved throughout this process, with phosphorylation and truncation playing significant roles^8^.

There are over 60 potential proteolytic cleavage sites on the tau protein that can lead to formation of a variety of fragments that are released into the interstitial fluid, cerebrospinal fluid and blood^9,10^. With the development of ultrasensitive technologies, it is now possible to detect tau in blood, providing a more accessible method for diagnosing AD. This facilitates the detection of early, pre-symptomatic stages of AD that would aid diagnosis and provide an early opportunity for therapeutic intervention. Detection of phosphorylated variants of tau have shown the best diagnostic performances to date. These include pTau181, pTau231 and pTau217^11–13^. These biomarkers are capable of distinguishing subjects on the AD continuum and those with other neurodegenerative disorders from cognitively unimpaired (CU) individuals with high diagnostic accuracy. Among them, pTau217 exhibits superior performance as a diagnostic and prognostic biomarker that can track longitudinal change in preclinical AD^13–15^.

Despite these advances, pTau biomarkers are specific to AD but are not necessarily reflective of structural tau pathology. Both pTau217 and pTau181 are highly correlated with amyloid pathology^13^. The field therefore still requires novel tau-based biomarkers that can address issues such as detection of early-stage tau pathology, diagnosis, disease staging and therapeutic monitoring^16^. A more comprehensive panel of biomarkers should improve our understanding of the disease and aid clinical decision making.

Several studies have shown that tau exists in CSF and plasma as a mixture of fragments containing N-terminal and mid-region (including proline region, R1 and R2) domains^17,18^. Current tau-based immunoassays, whether measuring total tau (T-tau) or phosphorylated tau, rely on capture and detection using monoclonal antibodies (mAbs), which recognise unique epitopes. This limits the number of tau fragments that can be detected and quantified.

In an attempt to overcome these limitations, we have made use of the potential of polyclonal antibodies (pAbs) and developed a well characterised proline region pAb which we term P.pAb. Using P.pAb, we have developed immunoassays that permit quantification of full-length tau as well as multiple tau fragments of varying size spanning the N-terminal and proline region comprising residues 113–251. Most tau fragments identified in CSF and plasma contain epitopes within this region^19^ that may or may not be phosphorylated. This proof-of-concept study suggests there is diagnostic value in utilising pAbs for measuring a larger repertoire of tau fragments available in plasma, as an alternative and potentially more appropriate approach to measuring total tau (T-tau).

**Fig. 1:**
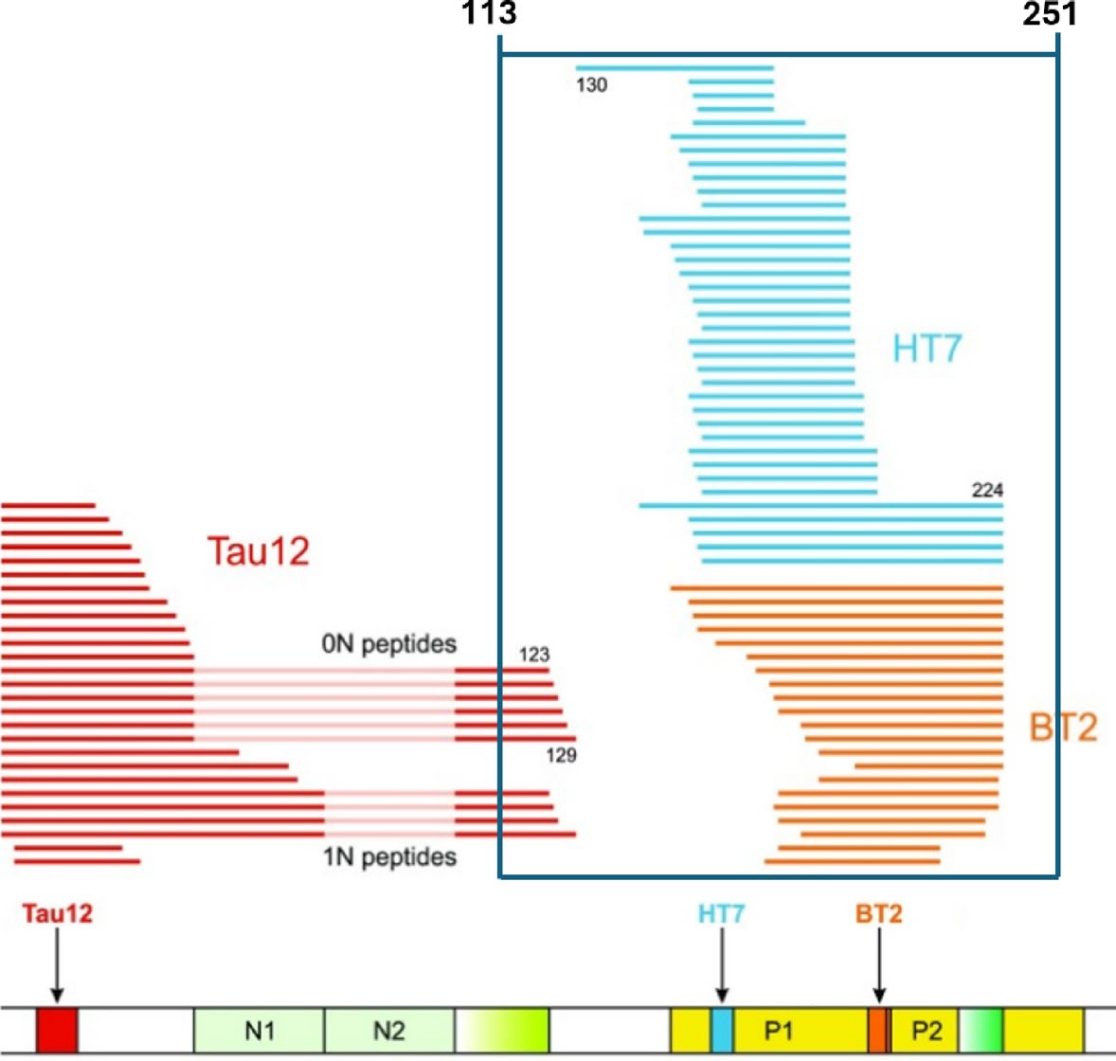
Endogenous tau species identified in CSF. Cicognola et al.^17^ performed IP-MS following capture by N-terminal (Tau12 aa 6–18) and proline region mAbs (HT7 aa 159–163 and BT2 aa 194–198). The blue box represents tau fragments that could be identified by P.pAb. Numbering and alignment are relevant to 2N tau isoforms. Regions not included in 0N and 1N isoforms have been dimmed. Figure adapted from^17^ under the terms of the Creative Commons Attribution 4.0 International License (http://creativecommons.org/licenses/by/4.0).

Current plasma T-tau assays are so named based on their ability to detect all six brain-specific tau isoforms. The term used, however, is a misnomer since it fails to report the total content of all tau fragments. Figure 1 summarises (and potentially still under-represents) the potential fragments that P.pAb can detect. The purpose of including the figure is simply to illustrate which tau fragments have previously been shown to exist in the literature. However, it is noteworthy that current mAb based T-tau immunoassays will miss all the fragments depicted in Fig. 1.

## Methods

### Immunisation of sheep using tau peptide 113–251

2N4R tau113-251 peptide (hereafter referred to as the 113–251 peptide) was expressed in bacteria and purified as previously described for 2N4R tau297-391^20^. For sheep immunisation, this peptide was used as the antigen with a primary inoculum (250 μg) followed by 4 subsequent boosts at 4-week intervals (125 μg). For immunisations, 113–251 peptide was mixed with Freund’s complete adjuvant and administered in a final volume of 2 mL. Serum was collected and used for the isolation and purification of P.pAb. Sheep immunisation and serum collection were performed by Ig Innovations Ltd. (Wales, United Kingdom). Experimental protocols involving sheep were reviewed and approved by Ig Innovations Ltd. All procedures were conducted in compliance with relevant national regulations governing the care and use of animals in research. This study is reported in accordance with the ARRIVE guidelines (https://arriveguidelines.org). Sheep serum samples were obtained from Ig Innovations Ltd. (Wales), where the animals were housed and immunised under standard husbandry conditions.

### Antigen-specific affinity purification

P.pAb was purified from sheep serum collected 22 weeks post-initial immunisation. The 113–251 peptide was diluted in PBS to 0.5 mg/mL and 2 mL was added to a chromatography column (Bio-Rad, #7321010), previously equilibrated with 3 mL PBS (pH 7.2). Subsequently, 1 mL AminoLink™ Plus coupling resin (Thermo Scientific, #20501) was added, followed by addition of 40 µL cyanoborohydride solution (Thermo Scientific #44892) and the mixture incubated overnight on a benchtop rotator at 4 °C with gentle rotation. The resin was then washed with 4 mL PBS and 4 mL 1 M Tris–HCl, after which 2 mL Tris–HCl and 40 µL cyanoborohydride solution were added. The column was incubated as before for 30 min and washed with 5 mL 1 M NaCl. Subsequently, 4.5 mL sheep serum and 0.5 mL 10 X PBS were added and the mixture incubated as before for 30 min. The serum addition and incubation steps were repeated, followed by 1 wash with 12 mL PBS. Antibodies were eluted with 8 mL 0.1 M glycine (pH 3) into 8 fractions each containing 50 µL 1 M Tris–HCl (pH 8). Eluted fractions were analysed by Sodium Dodecyl Sulphate—Poly-Acrylamide Gel Electrophoresis (SDS-PAGE) and the first 4 fractions combined. Amicon filter 50-kDa centrifuge columns (Amicon, #UFC505096) were used to perform PBS buffer exchange for 6 cycles. To derive a negative control pAb, protein A beads were used to isolate antibodies from serum of a sheep immunised with an unrelated antigen.

### Polyclonal serum binding ELISA

Clear Nunc Maxisorp™ 96-well plates were coated with equimolar concentrations of 22 nM 2N4R tau or 113–251 peptide and incubated for 1 h at 37 °C. The plates were washed 4 times with phosphate buffered saline (PBS) + 0.1% Tween-20 (PBS-T), blocked with PBS containing 2% non-fat milk (PBSM) for 1 h at 37 °C, and washed as before. Serum samples were diluted 1:4000 in PBSM and diluted 1:2 across the plate. The plates were incubated at room temperature for 1 h and washed as before. HRP-conjugated anti-sheep IgG (Sigma Aldrich, #A3415) was diluted 1:1000 in PBSM and added. Following incubation for 1 h at room temperature, the plates were washed as before and developed by the addition of 1-Step™ Ultra 3,3′,5,5′-Tetramethylbenzidine (TMB) Substrate Solution (Thermo Scientific, #34028). The reaction was stopped after 5 min with 50 µL 1 M H_2_SO_4_, and absorbance read at 450 nm.

### Binding ELISA

The binding profile of P.pAb to peptides and tau proteins was determined by ELISA as described above, with dGA (2N4R tau297-390) included as a negative control. P.pAb was used as the primary antibody and diluted 1:2 across the plate, from an initial concentration of 2 µg/mL (13 nM). Absorbance values read at 450 nm were used for the generation of antibody-binding curves fitted using a 4-parameter regression model (Prism version 9.5.1., GraphPad Software Inc., San Diego, USA) for the calculation of EC50 values. For in-house generated proline region binding CC7 mAb^21^, which was used to compare with P.pAb, secondary HRP-conjugated Anti-mouse IgG was used (Sigma Aldrich, #A6782).

### Epitope mapping

P.pAb epitopes were mapped by indirect ELISA against 25 overlapping peptides (13-aa in length) spanning the 2N4R tau103-259 region. Plates were coated with 5 µg/mL streptavidin (ThermoScientific, # 34302) and blocked as described above. Peptides were added (4 µg/mL) in PBS in duplicate. Subsequent antibody addition, plate development, and absorbance readings were conducted as described above, with the addition of primary antibodies at a concentration of 5 µg/mL.

### Surface plasmon resonance (SPR)

SPR was conducted using a Biacore X100 and HBS EP+ running buffer (Cytiva, BR100669). 2N4R tau was immobilised on the surface of a CM5 sensor chip via amine coupling, according to manufacturer’s guidelines. 2N4R tau113-251 and control pAbs were injected at five increasing concentrations (0.37 nM–90 nM) and a flow rate of 30 µL/min. Each cycle consisted of a 180 s association phase and a 420 s dissociation phase, followed by chip regeneration by a 30 s injection of 10 mM glycine pH 1.5 (Cytiva, BR-1003-54). Data were analysed using Biacore X100 evaluation software and fitted to a 1:1 binding model to ascertain pAb binding kinetics and dissociation constants.

### Immuno-electron microscopy

Sarkosyl-insoluble samples containing tau filaments were isolated from the frontal cortex of AD subjects as per previously published methods^22^. Filaments (3 μL) were placed onto the centre of a formvar/carbon-coated 400 mesh copper grid (EM Resolutions, # FC400Cu25) for 2 min. For all incubation steps, grids were placed on 25-μL drops. The grids were blocked for 10 min in blocking buffer (0.5% fish skin gelatine in PBS). Blocking was followed by overnight incubation at 4 °C. P.pAb or −ve control pAb (10 μg/mL) were diluted in blocking buffer. Grids were washed six times in blocking buffer, then incubated for 45 min in donkey anti-goat IgG conjugated to 10-nm colloidal gold particles (Abcam, #AB41496), diluted 1:25 in blocking buffer. Following incubation, the grids were washed six times with PBS, followed by a 5-min incubation on 2% glutaraldehyde in PBS. Fixation was followed by three 5-min washes in PBS and two 5-min washes in water. Finally, each sample was negatively stained by incubation on a drop of UranyLess EM Stain (Electron Microscopy Sciences, #22409) for 1 min. Samples were left to dry and then viewed under a JEOL 1400 plus transmission electron microscope with digital image capture at 25,000× magnification.

### Patient sample selection

Plasma samples (K2 EDTA anticoagulant) were available from an observational clinical study termed TRx-GTD-025 in which AD-diagnosed subjects and cognitively unimpaired (CU) healthy controls (Paired Volunteers) were monitored at baseline and 12 months later (post-baseline). All participants provided written informed consent prior to being enrolled in the study; if applicable, legal representatives provided consent on behalf of patients with reduced decision-making capacity. Subject consent for use of their biological samples for this research is covered by TRx-GTD-025 study- and country-specific informed consent forms. Only samples for which consent was obtained and not withdrawn, have been used in this research. The study was conducted in the United States of America, Canada and the United Kingdom; plasma samples from participants from all three countries are part of this research. Clinical assessments were performed based on National Institute on Aging (NIA) and Alzheimer’s Association (AA) criteria, in the absence of supporting biomarker evidence^23^. Based on our set criteria, post-baseline samples were selected from 43 AD and 45 CU individuals for our biomarker analyses (2 AD and 2 CU samples were not included in the pTau217-P.pAb analysis due to sample depletion). The criteria set for the AD group were: an MMSE score of 26 or below post-baseline with at least a two-point reduction from baseline over 12 months, consistent with clinical decline. Participants in the CU group had an MMSE of 30 at baseline and no reduction over 12 months. To evaluate the prognostic potential of the P.pAb-P.pAb assay, baseline samples from 13 AD patients who demonstrated clinical progression over 12 months (AD decliners) were compared to baseline samples from AD patients who remained stable (AD non-decliners) over the same period (Table 1).

**Table 1:** Demographics and cognitive scores of the selected patient population from TRx-GTD-025 study.

Patient group (n)	Mean age (SD)	MMSE score at baseline (SD)	MMSE score at 12 mo (SD)	ADAS-Cog_11_ at baseline (SD)	ADAS-Cog_11_ at 12 mo (SD)
AD (43)	70.1 (9.0)	23.5 (2.1)	19.1 (3.2)	19.5 (6.3)	22.1 (8.6)
CU (45)	68.4 (9.3)	29.3 (0.9)	30 (0)	4.8 (2.1)	4.8 (2.3)
AD decliners (13)	72.7 (7.1)	23.3 (1.8)	18.7 (3.0)	21.2 (6.7)	28.8 (8.5)
AD non-decliners (17)	72.5 (9.0)	23.5 (2.1)	25.3 (2.5)	13.8 (6.3)	10.7 (7.2)

Participants on this study were assessed at baseline and at 12 month follow-up. SD, standard deviation; AD, Alzheimer’s disease; CU, cognitively unimpaired; MMSE, mini-mental state examination; ADAS-Cog_11_, Alzheimer’s Disease Assessment Scale–Cognitive Subscale. Post-baseline samples used for AD and CU. Baseline samples used for AD decliners and AD non-decliners.

### Simoa assays

#### P.pAb-P.pAb assay

Preparation and activation of P.pAb paramagnetic capture beads and biotinylation of P.pAb detector were performed according to Quanterix instructions. Calibrator curves were run in duplicate by threefold dilutions of 2N4R tau (540–0.74 pg/mL). The analytical LLOQ of the assay was determined to be 0.74 pg/mL (avg. blank + 3 × SD). Plasma samples were diluted 1:4 and analysed in duplicate. All dilutions were performed using Tau 2.0 diluent (Quanterix, #103847). A capture bead mixture, containing a 50:50 ratio of P.pAb capture beads to blocked helper beads (Quanterix #103208), was added. The Simoa 3-step protocol was followed: samples were first incubated with capture beads for 30 min, biotinylated P.pAb detector (150 ng/mL) for 20 min, then with Streptavidin-β-Galactosidase (150 pM) for 10 min, with wash steps in between. All further steps were performed in accordance with the manufacturer’s instructions. This assay was validated as described in the supplementary and Supplementary Figure S5.

#### pTau217-P.pAb assay

Biotinylated P.pAb detector (150 ng/mL) was used in combination with capture beads from the ALZpath Simoa pTau217 advantage v2 kit (Quanterix #104371, lot #999036). The calibrator curve was run in singlicate by diluting the kit calibrator twofold (400–25 pg/mL) and three blank wells were assessed to derive the analytical LLOQ. The analytical LLOQ of the assay was determined to be 25 pg/mL (avg. blank + 3 × SD). All dilutions were performed using diluent provided in the kit. Plasma samples were diluted 1:4 and analysed in singlicate. The Simoa 2-step protocol was followed, and all further steps were performed in accordance with the manufacturer’s instructions. The assay was validated as described in the supplementary and Supplementary Figure S6.

#### Commercial Simoa assays

Quanterix ALZpath pTau217 V2 kit (#104371, lot #999036), NF-light V2 advantage kit (#104073, lot #503836), Tau advantage kit (#101552, lot #504322) and Neurology 3-plex A kit (#101995, lot #502238) were used to quantify pTau217, neurofilament light (NfL), T-tau and T-tau + Aβ42 + Aβ40 respectively, in selected plasma samples.

### Statistical analysis

All results are expressed as mean ± SEM, unless otherwise stated. For statistical analysis, an unpaired t-test was performed. Normality of data distributions was assessed prior to group comparisons. For biomarkers with normally distributed data (P.pAb and pTau217-Tau12 assays), unpaired t-test was performed, applying Welch’s correction where variances were unequal. For biomarkers that did not follow a normal distribution, non-parametric Mann–Whitney U test was performed. All tests were two-tailed, and statistical significance was set at *p* < 0.05. Correlation analysis was performed by Spearman’s rank correlation. All statistical analyses and determination of AUC were performed on Prism version 9.5.1. (GraphPad Software Inc., San Diego, USA).

## Results

### Immunoreactivity of sera

**Fig. 2:**
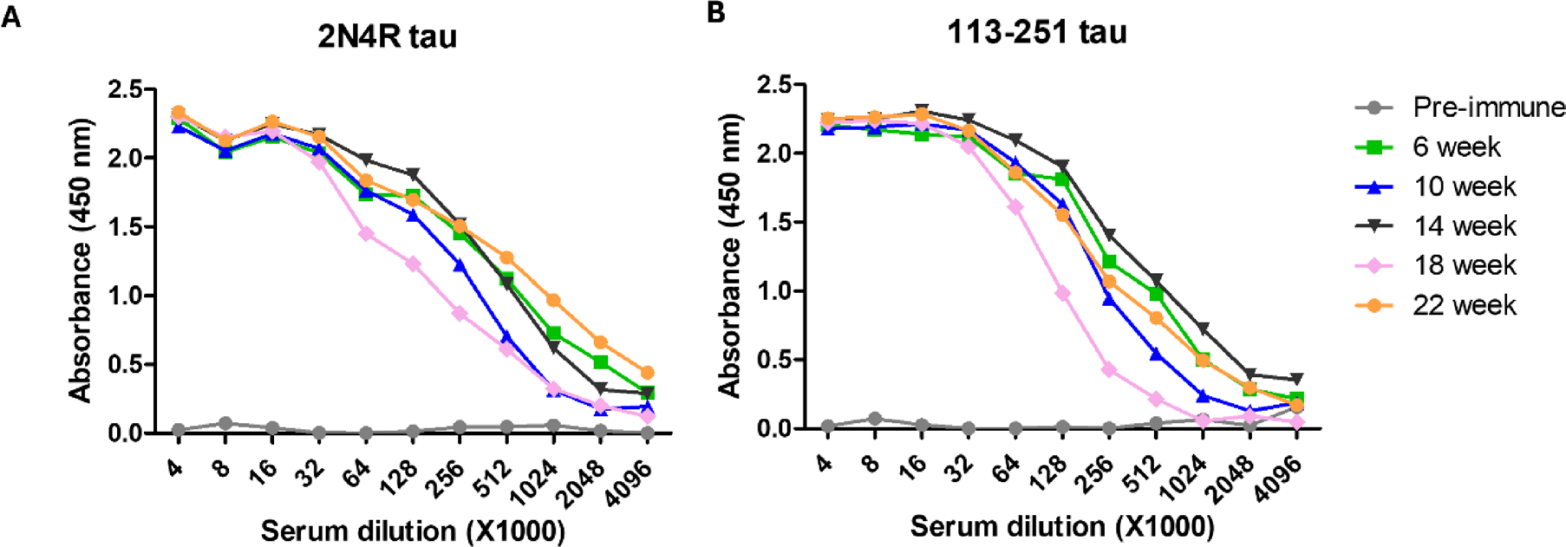
Antigen-specific immune responses for sera obtained from sheep following hyperimmunisation with tau peptide 113–251. Five rounds of immunisation were delivered at 4-week intervals with serum collected 10–14 days post-boost. Immune responses were analysed by ELISA with surface-bound antigens (**A**) 2N4R tau and (**B**) tau peptide 113–251, each at a concentration of 21.8 nM and polyclonal sera from a starting dilution of 1:4000. Pre-immune signals were similar to control PBSM wells. Average signals derived from *n* = 4 and *n* = 3 experiments for (**A**) and (**B**), respectively.

Hyperimmunisation of a Welsh-bred sheep with tau peptide 113–251 generated an antigen-specific immune response. This was determined by assessing binding of antibodies to full-length 2N4R tau and tau peptide 113–251 in ELISA using polyclonal sera collected pre-immunisation and 10–14 days following each boost. Immunoreactivity to both antigens was observed and this appeared to show saturation after the initial immunisation (Fig. 2). The serum collected after 22-weeks was used for generation of affinity-purified P.pAb.

### Immunoreactivity of P.pAb

**Fig. 3:**
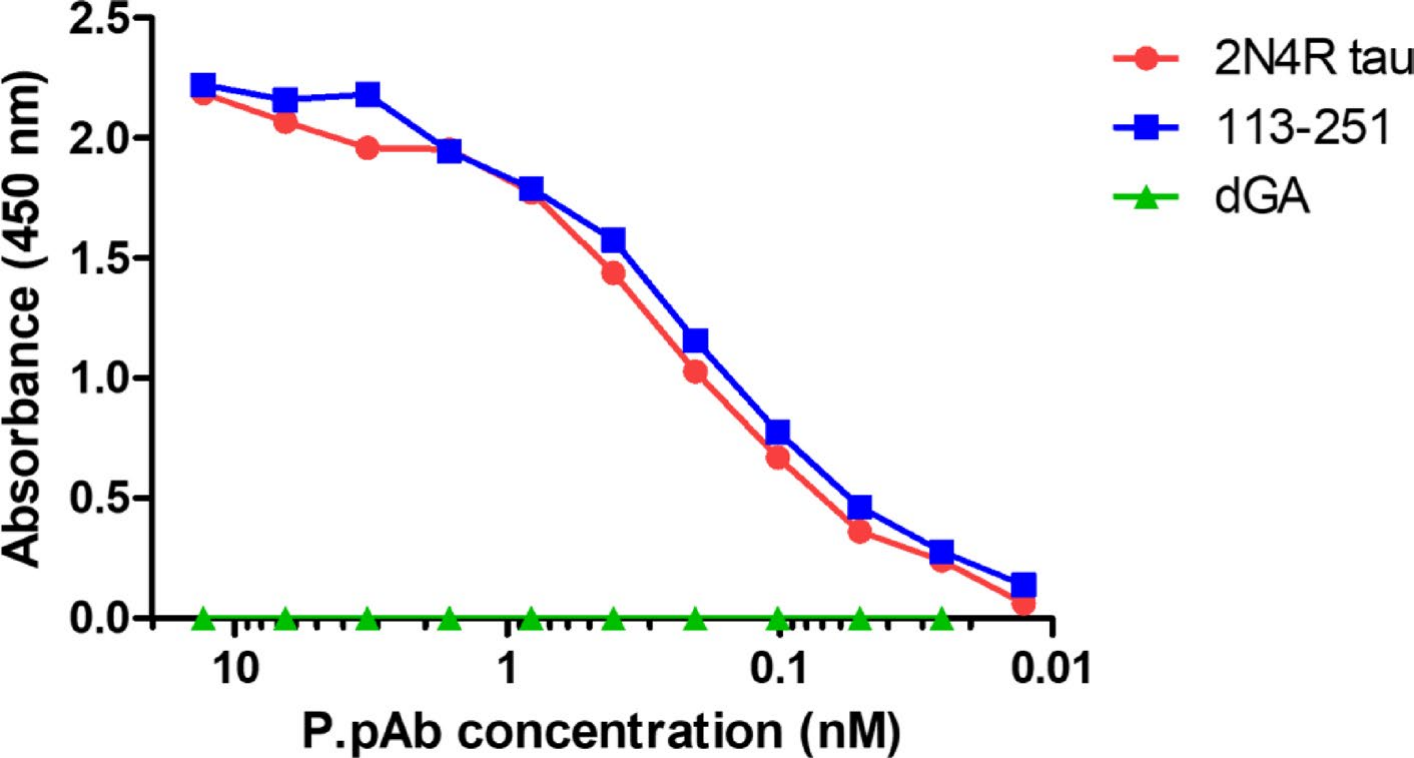
Binding profile for P.pAb to full length tau and tau fragment 113–251. Equimolar concentrations (21.8 nM) of 2N4R tau, tau peptide 113–251 and a negative control (dGA; tau297-390) were utilised. P.pAb was diluted twofold across the ELISA plate at a starting concentration of 13.33 nM. The experiment was performed in quadruplicate. Error bars were negligible and not included in the graph for display purposes.

Binding of affinity-purified P.pAb (13.3 -0.01 nM) with equimolar concentrations of 2N4R tau, tau peptide 113–251, and tau peptide 297–390 (dGA, -ve ctrl) was determined by indirect ELISA (Fig. 3). P.pAb binding to dGA was absent with similar half maximal effective concentrations (EC_50_) to 2N4R tau and tau peptide 113–251 (0.14 nM and 0.09 nM, respectively). On comparison with an in-house generated proline-binding mAb (named CC7, aa 145–157 epitope^21^), P.pAb showed 7.2-fold improvement in binding to 2N4R tau and 6.3-fold improvement in binding to tau peptide 113–251, based on determined EC_50_ (data summarised in Supplementary Table S2). Additionally, SPR analysis shows a fast on rate and slow off rate, suggesting high affinity binding to 2N4R tau, likely due to multiple antibodies binding to multiple epitopes (Supplementary Figure S1). Furthermore, relevance of P.pAb to AD was confirmed by assessing its binding to paired helical filaments isolated without protease treatment from human AD frontal cortex tissue via immuno-EM (Supplementary Figure S2).

### Epitope mapping of P.pAb

**Fig. 4:**
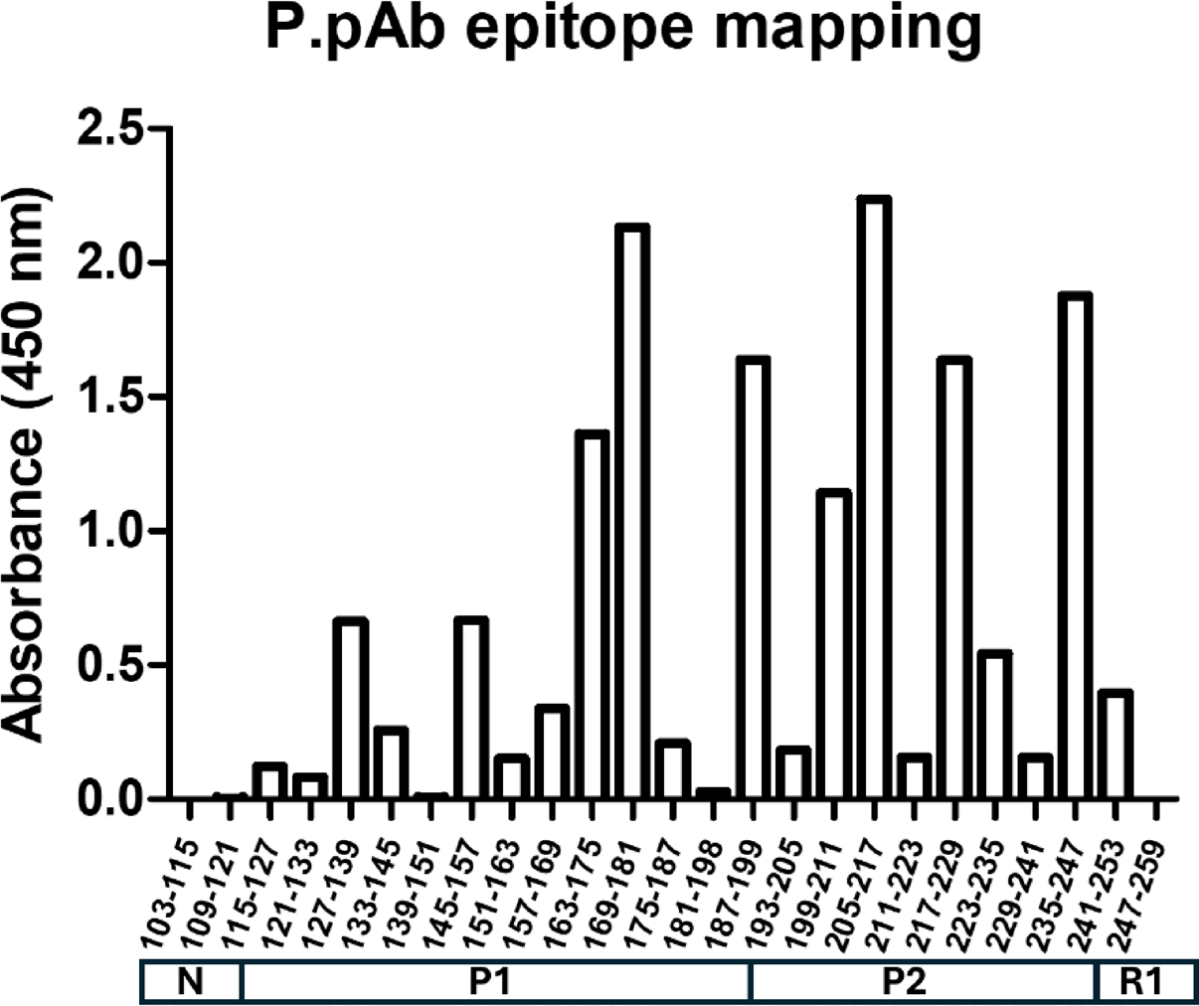
Binding interactions of P.pAb with overlapping 13-aa peptides spanning 2N4R tau103-259. Binding of a saturating concentration of antibody (33.3 nM) to surface-bound peptides was measured. A depiction of the tau protein is included to show the region of the protein in which these peptides reside. N = N-terminal end of fragment, P1 and P2 = Proline region domain subdivisions, R1 = beginning of microtubule-binding domain repeat 1.

Since the purpose of generating this pAb was to detect multiple fragments of the tau protein in immunoassays, the binding of P.pAb to truncated tau fragments was assessed. P.pAb displayed strong binding to 2N4R tau peptides 1–231, 113–201, 1–155 and 186–391, with no binding to a negative control fragment (297–391; data not shown). Further epitope mapping was performed by indirect ELISA using tau peptides consisting of 13-aa overlapping sequences, that span the sequence 103–259. Binding to peptides spanning the full domain was observed, with signal intensity differing between peptides (Fig. 4). This confirmed binding of P.pAb to various tau epitopes spanning residues 113–251.

### Selection of TRx-GTD-025 study plasma samples

For the ‘proof-of-concept’ analysis, plasma samples from 45 CU individuals and 43 AD patients that met our set criteria (Methods section) were included in the study population. This provided an initial proof-of-concept biomarker cohort comprising a suitable number for analysis. Based on MMSE scores at baseline and at 12-month follow-up, the AD sample cohort can be categorised into four groups (Table 1). (a) 12 month samples of Mild AD (19 patients; MMSE = 21–26) and moderate AD (24 patients; MMSE = 10–20) AD; (b) 12 month CU samples (45 patients; MMSE of 30 at 12 months; (c) a cohort of 17 baseline samples from AD patients that showed clinical decline at 12-month follow-up (AD decliner group) and (d) a cohort of 17 baseline samples from AD patients who showed no clinical decline at 12-month follow-up (AD non-decliner group).

### Plasma biomarker assessment of samples using commercial kits

Since there was no corresponding imaging or fluid biomarker assessment available for the patient sample cohort, we chose to validate the plasma samples biologically, using commercially available Simoa kits to quantify the levels of NfL and pTau217. We refer to the pTau217 assay as pTau217-Tau12. Since Tau12 recognises tau9-16, this assay reflects the tau9-217 fragment.

**Fig. 5:**
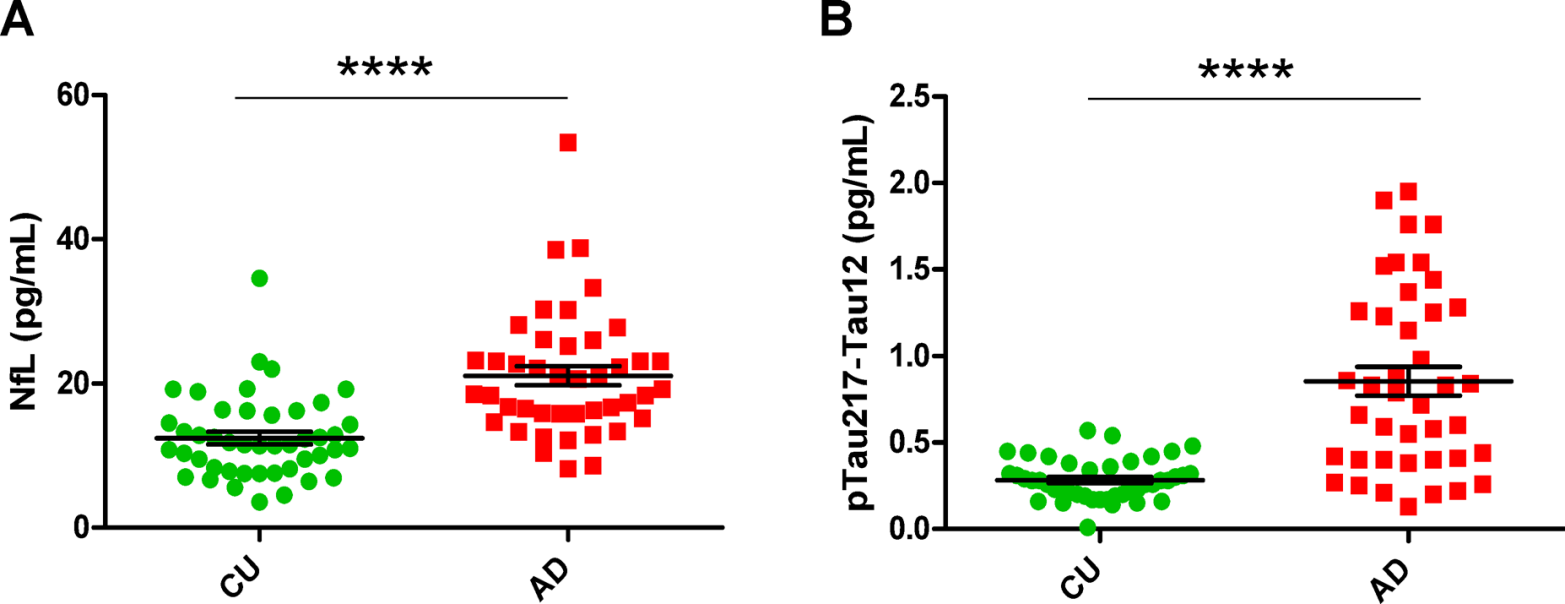
Measured levels of (**A**) NfL and (**B**) pTau217-Tau12 in patient samples. Graphs show the distribution of results in CU and AD groups. Quanterix NfL advantage V2 and ALZpath pTau217 kits were used to quantify levels of biomarkers. CU, cognitively unimpaired; AD, Alzheimer’s disease; NfL, neurofilament light. Mann–Whitney U test (**A**) and unpaired t-test with Welch’s correction (**B**) performed for statistical analyses. **** = *p* < 0.0001. Values expressed as mean ± SEM.

Mean NfL concentrations were 1.6-fold higher in the AD group when compared to the CU group (20.8 ± 1.3 pg/mL and 12.5 ± 0.8 pg/mL, respectively, *p* < 0.0001, Fig. 5 A). Mean pTau217-Tau12 concentrations were threefold higher in the AD group when compared to the CU group (0.84 ± 0.08 pg/mL 0.28 ± 0.02 pg/mL, respectively, *p* < 0.0001, Fig. 5B). There was a significant positive correlation between these two biomarkers (Fig. 7 F, r = 0.59, *p* < 0.0001), consistent with AD-related neurodegeneration in this cohort. Standard curves for these assays are displayed in Supplementary Figure S3A and S3 B.

### Plasma biomarker assessment in two P.pAb assays

**Fig. 6:**
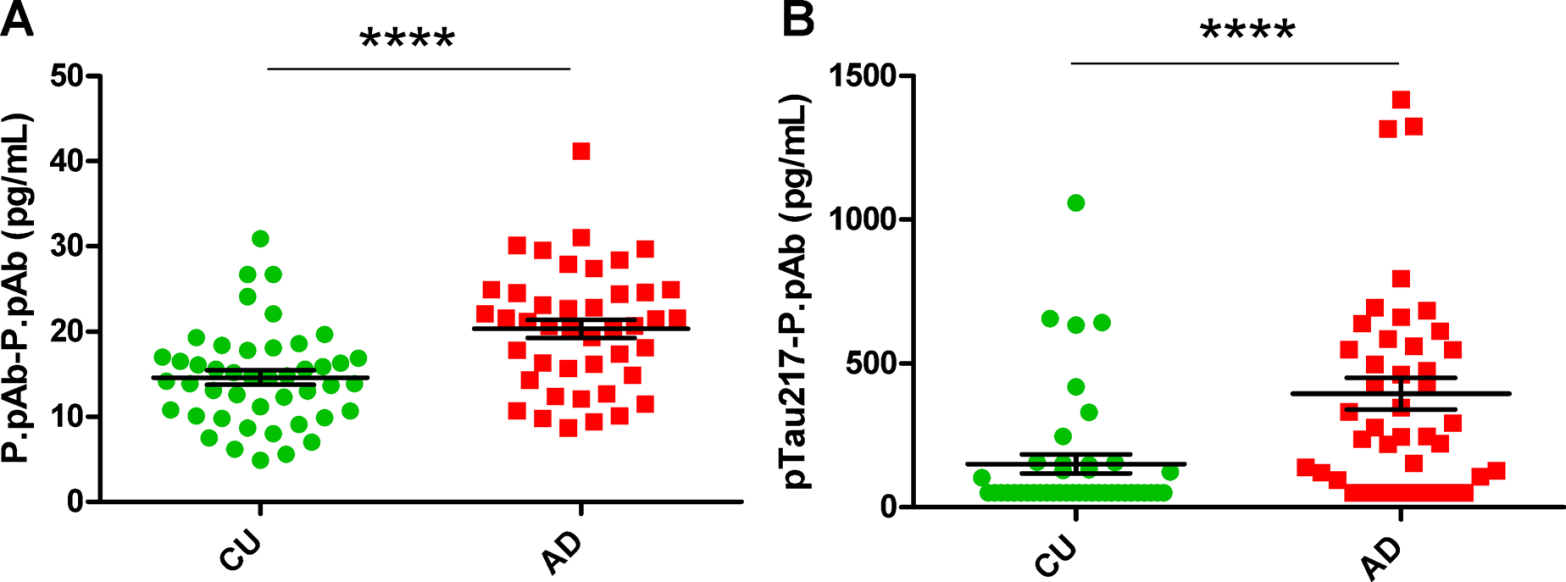
Measured levels of (**A**) P.pAb-P.pAb and (**B**) pTau217-P.pAb in selected patient samples. Graphs show the distribution of results in CU and AD groups. CU, cognitively unimpaired; AD, Alzheimer’s disease. Unpaired t-test (**A**) and Mann–Whitney U test (**B**) performed for statistical analysis. **** = *p* < 0.0001. Values expressed as mean ± SEM.

P.pAb was used as the detector antibody and paired with itself for capture to maximise the detection of tau fragments containing epitopes within the 2N4R tau113-251 region. This assay is referred to as P.pAb-P.pAb. Mean P.pAb-P.pAb plasma concentrations were 1.4-fold higher in the AD group when compared to CU (20.2 ± 1.1 pg/mL and 14.7 ± 0.8 pg/mL, respectively, *P* < 0.0001, Fig. 6A). To confirm that the signals generated reflect the abundance of tau, assays were performed with a negative control pAb. P.pAb in combination with this negative control pAb as detector and vice versa, as capture, yielded no standard curve or quantification in plasma samples (Supplementary Figure S4).

In a second assay, pTau217 was used as the capture antibody with P.pAb as the detector. For this assay, ALZpath pTau217 kit components were used, with exception of the detector, which was replaced by biotinylated P.pAb. This assay will be referred to as pTau217-P.pAb. Mean pTau217-P.pAb plasma concentrations were 2.8-fold higher in the AD group when compared to CU (375.8 ± 51.2 pg/mL and 149 ± 32.2 pg/mL, respectively, *p* < 0.001, Fig. 6B). It must be noted that due to the relatively low sensitivity of the pTau217-P.pAb assay, 28 samples in the CU group had values below the analytical LLOQ of 25 pg/mL for the assay. This was also true for eight of the AD samples. These 36 samples were assigned a value of 50 pg/mL, based on the following equation: (LLOQ ÷ 2) × dilution factor. Standard curves for these assays are displayed in Figure S3 C and S3 D.

### Correlation analyses for assays

**Fig. 7:**
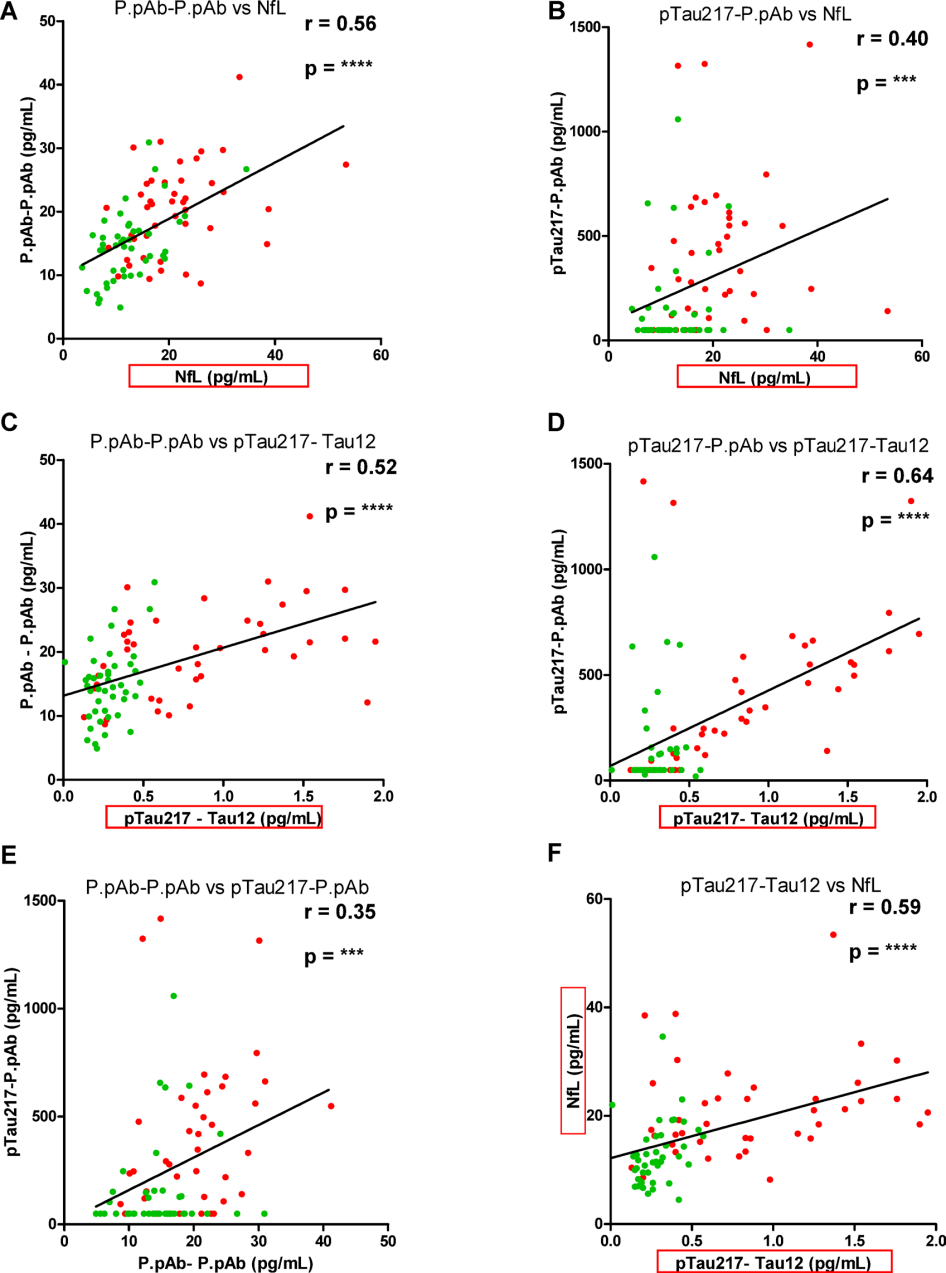
Correlation analyses of assays. (**A**) P.pAb-P.pAb vs NfL. (**B**) pTau217-P.pAb vs NfL. (**C**) P.pAb-P.pAb vs pTau217-Tau12. (**D**) pTau217-P.pAb vs pTau217-Tau12. (**E**) P.pAb-P.pAb vs pTau217-P.pAb. (**F**) pTau217-Tau12 vs NfL. Commercial assays have been highlighted with a red box. AD samples are in red and CU samples are in green. *r* = Spearman’s correlation coefficient. *p* < 0.0001 (****), *p* < 0.001(***).

There was a significant positive correlation between the P.pAb-P.pAb assay and both NfL (Fig. 7A, *r* = 0.56, *p* < 0.0001) and pTau217-Tau12 (Fig. 7C, *r* = 0.52, *p* < 0.0001). Likewise, there was a significant positive correlation of the pTau217-P.pAb assay with NfL (Fig. 7B, *r* = 0.40, *p* = 0.0002) and pTau217-Tau12 (Fig. 7D, *r* = 0.64, *p* < 0.0001). Furthermore, both P.pAb assays showed a significant positive correlation with each other (Fig. 7E, *r* = 0.35, *p* = 0.0009).

### Assessing the diagnostic accuracy of assays

**Fig. 8:**
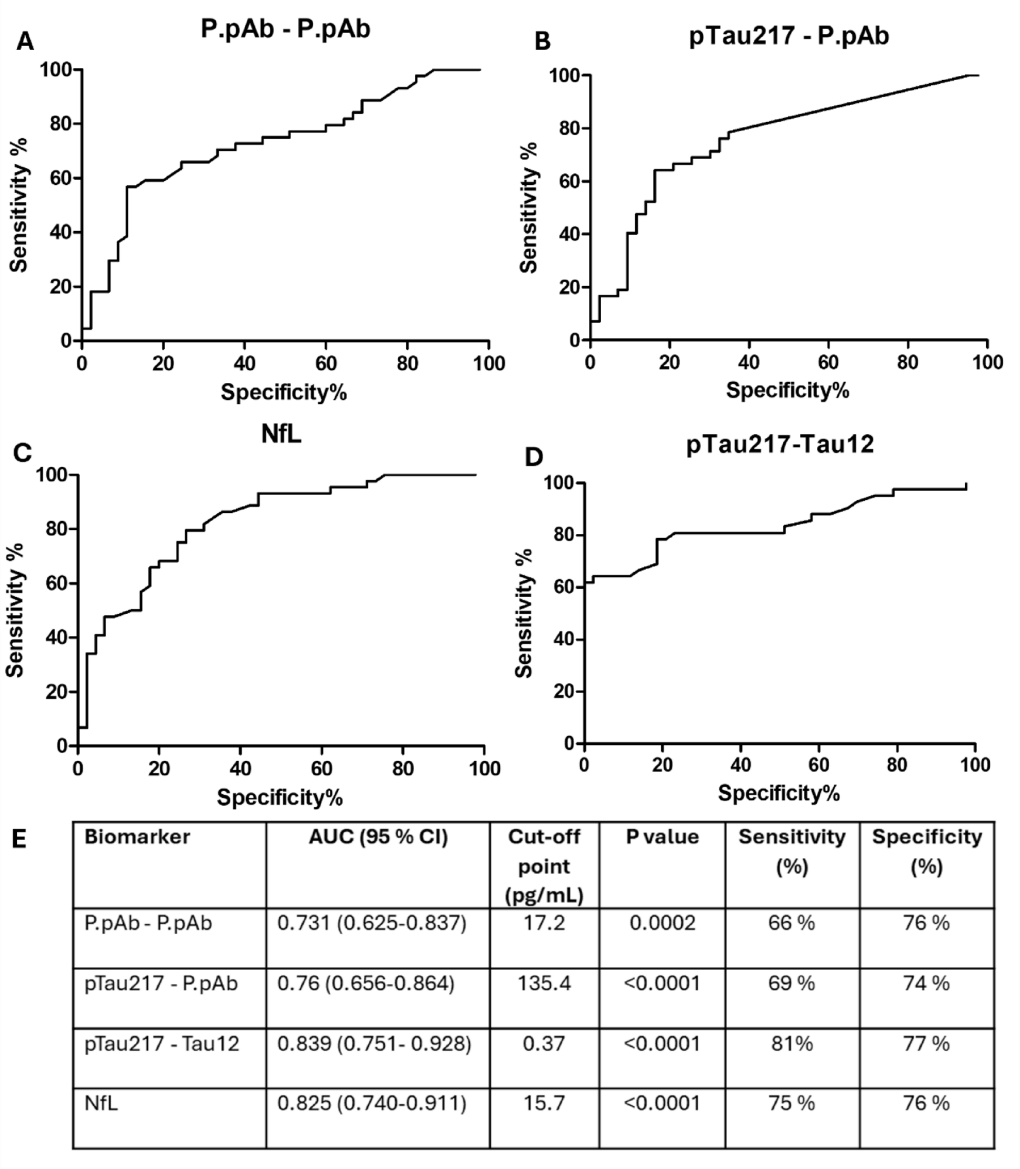
ROC curves showing the sensitivity and specificity of the biomarkers for discriminating AD from CU. (**A**) P.pAb-P.pAb, (**B**) pTau217-P.pAb, (**C**) NfL, and (**D**) pTau217-Tau12 in differentiating AD from CU in the selected cohort. (**E**) Table summarising the findings. Optimal cut-off points maximising sensitivity and specificity are reported.

Receiver operating characteristic (ROC) analyses were performed to assess the performance of P.pAb assays in differentiating between CU and AD groups. This was also performed for NfL and pTau217-Tau12 biomarkers. While all biomarkers showed good accuracy, as summarised in Fig. 8, the commercially available pTau217-Tau12 and NfL assays displayed better performance (AUC = 0.839 and 0.825, respectively) than the P.pAb-P.pAb and pTau217-P.pAb assays (AUC = 0.731 and 0.760, respectively).

### Assessing diagnostic accuracy for sub-categories of the AD group

Because clinical diagnosis may only be 73% accurate^24,25^, it is possible that some of the AD population may have been misdiagnosed. The lower AUC of the pTau217-Tau12 assay compared with the literature further suggests that this may be the case. To refine the study population further, subjects were subcategorised based on a pTau217-Tau12 concentration threshold of > 0.63 pg/mL. This cut-off point was determined by Ashton et al. following assessment of three large patient cohorts using the same ALZpath pTau217 kit that was also utilised in our study^13^. It is worth noting that the cut-off established by Ashton et al. was derived using a different kit lot. However, pTau217 kit lot-to-lot variability has been evaluated across three ALZpath pTau217 kit lots and shown to be within ± 15%^26^. None of the CU samples were above this cut-off point, however 18 AD samples were below the cut-off point and the data for these samples were removed for this sub-analysis.

**Fig. 9:**
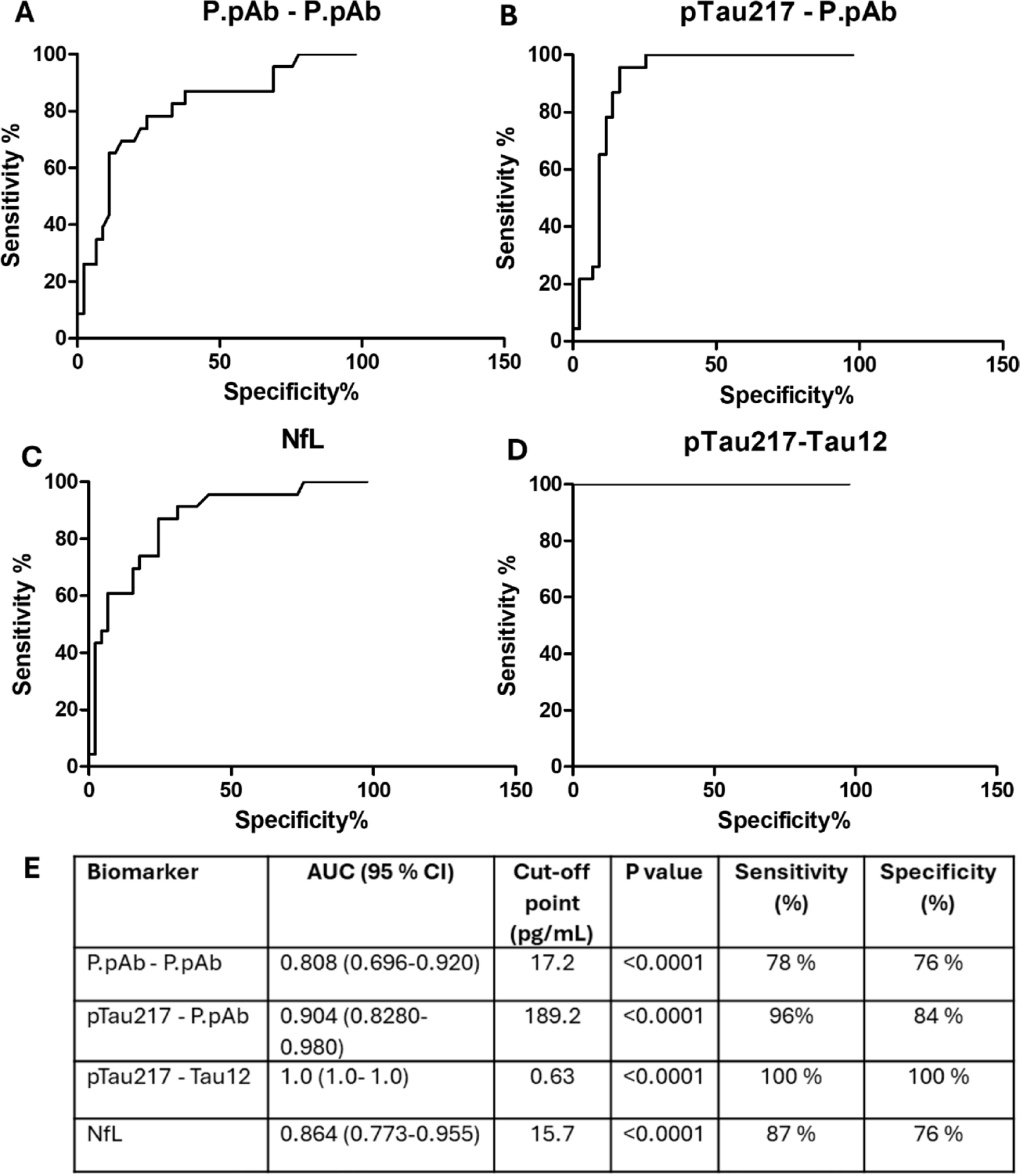
ROC curves showing the sensitivity and specificity of the biomarkers for discriminating AD from CU following sub-categorisation of the AD group. Only those AD samples above 0.63 pg/mL pTau217-Tau12 were included (*n* = 25). (**A**) P.pAb-P.pAb (**B**) pTau217-P.pAb (**C**) NfL and (**D**) pTau217-Tau12. (**E**) Table summarising the findings. Optimal cut-off points maximising sensitivity and specificity are reported.

Based on this sub-analysis of 23 AD samples, the commercial pTau217-Tau12 assay has an AUC of 1.0 and 100% sensitivity and specificity. The NfL assay shows an improved AUC, increasing from 0.825 (95% CI: 0.740–0.911) to 0.864 (95% CI: 0.774–0.955). Sensitivity for the NfL assay was also increased by 12%, rising from 75 to 87%, with specificity remaining unchanged (76%). The P.pAb-P.pAb assay shows an improved AUC, increasing from 0.731 (95% CI: 0.625–0.837) to 0.808 (95% CI: 0.696–0.920). Sensitivity was also increased by 12%, rising from 66 to 78%. The pTau217-P.pAb assay shows an improved AUC, increasing from 0.760 (95% CI: 0.656–0.864) to 0.904 (95% CI: 0.8280–0.980). Sensitivity and specificity were also increased by 27% (from 69 to 96%) and 10% (from 74 to 84%), respectively (Fig. 9). Improvements in assay parameters are summarised in Supplementary Table S3.

### P.pAb-P.pAb assay outperforms commercial T-tau assay

**Fig. 10:**
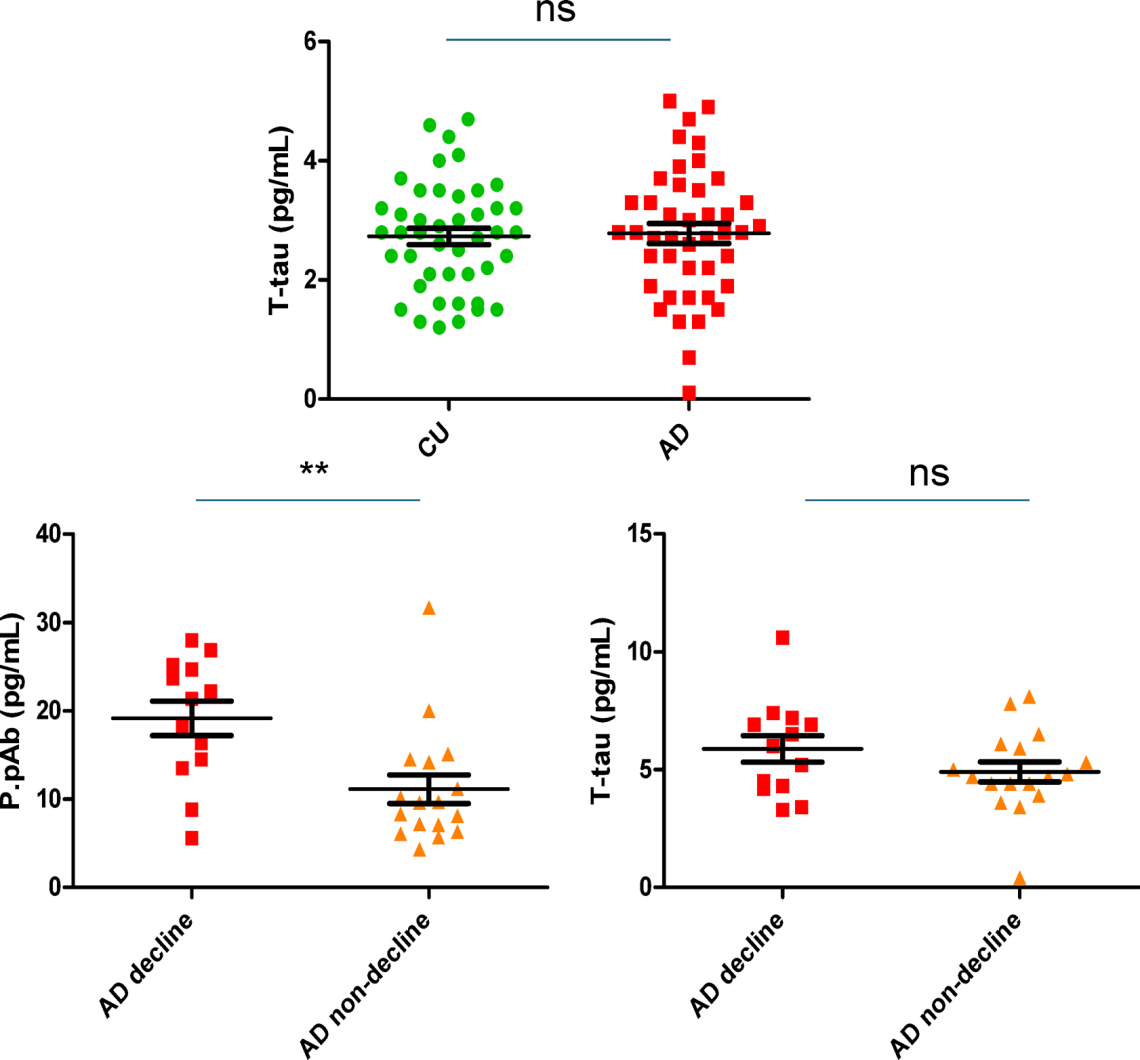
Assessing the performance of T-tau for comparison with P.pAb-P.pAb assay: (**A**) Quanterix tau advantage kit (**B**) P.pAb-P.pAb assay (**C**) Quanterix neurology 3-plex A kit were utilised. 12-month samples were assessed in (**A**). Baseline samples were assessed in (**B**) and (**C**). Graphs show the distribution of results in CU, AD, AD decliner and AD non-decliner groups. CU, cognitively unimpaired; AD, Alzheimer’s disease; ns, non-significant. Unpaired t-test (**A**) and Mann–Whitney U test (**B**) and (**C**) was performed for statistical analysis. ** = *p* < 0.0061. Values expressed as mean ± SEM. (**A**) *p* = 0.82 (**B**) *p* = 0.0061 (**) (**C**) *p* = 0.172.

Since the P.pAb-P.pAb assay is essentially a T-tau assay, capable of detecting a greater number of tau fragments, the performance of this assay was compared with that of a commercially available T-tau kit, which uses a combination of mAbs that target epitopes that cover the N-terminal to mid-domain (R1) of tau, and is able to recognise all six known splice variants. The T-tau assay failed to show any differentiation between CU and AD groups (2.7 ± 0.14 pg/mL and 2.8 ± 0.17 pg/mL, respectively, *p* = 0.82, Fig. 10A). Standard curve for this assay is displayed in Figure S3 E.

Furthermore, P.pAb-P.pAb and T-tau were quantified for a subset of baseline plasma samples from AD decliners (*n* = 13) and AD non-decliners (*n* = 17), based upon cognitive decline over a 12-month period. Mean P.pAb-P.pAb plasma concentrations were 1.7-fold higher in the AD decliner group compared to the AD non-decliner group (19.2 ± 2.0 pg/mL and 11.1 ± 1.6 pg/mL, respectively, *p* = 0.0035, Fig. 10B). T-tau concentrations did not reach statistical significance, suggesting this assay could not differentiate between AD decliners and AD non-decliners (4.9 ± 0.4 pg/mL and 5.9 ± 0.6 pg/mL, respectively, *p* = 0.172, Fig. 10C). however, the possibility that this reflects limited sample size and statistical power cannot be excluded.

## Discussion

Several paired antibody assays claim to quantify ‘'total’ tau levels^27^. Although these assays can detect all six tau isoforms, they overlook information regarding truncated tau species which could provide valuable information. This limitation stems from their reliance on mAbs that bind to a single epitope within the tau protein, thereby missing smaller fragments that could be produced by over 60 cleavage sites within the tau molecules. Consequently, despite showing increased concentrations in AD samples, there is a large overlap between CU and AD plasma samples, limiting their utility as biomarkers for AD^28^. Furthermore, T-tau is regarded as a biomarker of neuronal damage that is not specific for AD^29–31^.

Therefore, T-tau cannot be categorised as a biomarker specific for tau pathology. CSF T-tau was categorised as a biomarker of neurodegeneration in the National Institute on Aging and Alzheimer’s Association (NIA-AA) 2018 framework^32^, and in the updated 2024 criteria^33^, it is considered diagnostically useful only when assessed in combination with Aβ42 in CSF as a ratio (T-tau/Aβ42). Current CSF T-tau assays show abnormalities five years prior to symptom onset, whereas plasma pTau181 and pTau217 levels show abnormalities over twenty years prior to symptom onset^34^. Given these current limitations and since tau plays a critical role in AD, exploring alternative approaches to measuring tau in plasma could provide a more accurate and clinically relevant measure that could be translated to improved diagnostic tests.

We have developed Simoa assays using P.pAb that are capable of distinguishing AD from CU plasma samples whether it is paired with itself or with a pTau217 mAb to capture tau in plasma. The latter is considered to be a more specific and informative biomarker of AD since P.pAb-P.pAb can still detect peripheral sources of tau. In the initial assessment using all samples in this study, the AUC values of P.pAb-P.pAb and pTau217-P.pAb were 0.73 (sensitivity = 66%, specificity = 76%) and 0.76 (sensitivity = 69%, specificity = 74%), respectively. Although these assays demonstrated relatively good performance for distinguishing CU participants from AD patients, sample selection may have resulted in a reduced performance of these assays since AD samples were based solely on clinical diagnoses, with no histopathological or biomarker assessment. It has been reported that, based on clinical assessment alone, specialist physicians diagnose AD with 73% accuracy^24,25^. A separate study reported a specificity of only 23% when using the National Institute of Neurological and Communicative Disorders and Stroke-Alzheimer’s Disease and Related Disorders Association (NINCDS-ADRDA) criteria, due to misdiagnosing 20 out of 48 frontotemporal dementia patients as having AD, determined following post-mortem assessment^35^.

An AD sub-population, following assessment of pTau217 levels in plasma was identified using a cut-off value of greater than 0.63 pg/mL in the pTau217-Tau12 assay. Twenty-three AD samples met this criterion, suggesting some of the others could have been misdiagnosed. None of the CU samples exceeded this cut-off point. This cut-off point was determined by Ashton et al. following assessment of three large patient cohorts and corresponds to the upper boundary of a three-range model for plasma pTau217. Specifically, values exceeding 0.63 pg/mL were associated with 95% specificity for Aβ-positivity, as measured by PET imaging or CSF analysis, thereby increasing confidence in identifying individuals likely to exhibit underlying amyloid pathology^13^. This sub-population analysis resulted in much improved accuracies of all assays; P.pAb-P.pAb, AUC = 0.81 (sensitivity = 78%, specificity = 76%), pTau217-P.pAb, AUC = 0.90 (sensitivity = 96%, specificity = 84%). Eight AD samples in the pTau217-P.pAb analysis were below the LLOQ of the assay and were unquantifiable. These were also below the 0.63 pg/mL cut-off point as quantified by the commercial pTau217-Tau12 assay, which provided additional evidence that the pTau217-Tau12 and pTau217-P.pAb assays are in agreement. In contrast to these findings, the commercial T-tau assay could not differentiate between the CU and AD groups in our study. This suggests that the P.pAb-P.pAb assay may be a more disease-relevant assay for measuring tau protein in plasma. It should however be noted that other factors, including differences in assay sensitivity, antibody affinity, as well as epitope recognition, may contribute to the observed performance, and further validation and head-to-head assay comparisons are required to clarify the extent to which fragment detection contributes to the performance differences observed. It is well recognised that tau metabolism is altered in different dementias. Therefore, another potential application of P.pAb is in distinguishing between different tauopathies and other disorders of tau dysfunction, with differential truncation patterns being reported for progressive supranuclear palsy^36^, corticobasal degeneration^37^ and traumatic brain injury^38^. This is further summarised by Quinn et al.^39^.

### Limitations

This proof-of-concept study shows the utility of a diagnostic assay that has the potential to detect multiple tau fragments in plasma, in addition to full length tau, highlighting its utility as a biomarker for AD. There are however limitations which must be addressed in future work. Firstly, a more comprehensive analysis using a cohort of validated AD plasma samples is needed to confirm these findings. Furthermore, to overcome the low sensitivity of the pTau217-P.pAb assay, we would ensure a lower sample dilution is used. In addition, caution should be taken in interpreting the absolute values reported, since the calibrator material utilised is not optimal. For the pTau217-P.pAb assay, the calibrator that is part of the ALZpath pTau217 kit was used. Details of this calibrator are not known, and this could be a reason for the relatively high LLOQ of the assay and possibly, the high level of quantification. If the high levels of signal generated prove to be true, this approach could potentially eliminate the need for ultrasensitive technologies. Further experiments are required to determine if this assay can be transferred to more accessible methods such as ELISA and Western blot. Additionally, we do not currently have direct evidence of quantifying the relative proportions of full-length versus fragmented tau in our samples. Future studies employing IP-MS, will be necessary to disentangle these contributions and more precisely define the molecular species detected. To overcome the problems associated with pAbs as diagnostic agents, particularly regarding batch-to-batch consistency^40^, a mixed panel of mAbs targeting the proline region of tau could offer a solution. By using different combinations of such mAbs with known epitopes, we can enhance our knowledge of the plasma-tau profile and develop a better understanding of which fragments are best quantified for the greatest discrimination between healthy people and those in the early stages of developing AD.

## Conclusion

We have generated an affinity-purified pAb that binds to epitopes across the proline-rich region of tau protein. This was used to develop two Simoa assays, P.pAb-P.pAb and pTau217-P.pAb, that were specific for tau and demonstrated robust analytical performance. These assays discriminated between CU and AD samples. While they did not outperform the pTau217-Tau12 assay in the selected sample cohort, the P.pAb-P.pAb assay may have other applications, including monitoring disease progression, response to treatment and distinguishing between tauopathies. The pTau217-P.pAb assay generated a high level of signal, likely due to the combination of effective capture of pTau217 species and the cumulative detection by multiple detector antibodies binding to multiple tau fragments. This level of signal presents the potential for a simple AD test that does not require ultrasensitive detection technology. The P.pAb-P.pAb assay was able to differentiate between baseline plasma samples from patients who exhibited clinical decline at 12-month follow-up compared to those who remained stable, suggesting it also has potential prognostic utility. Finally, by outperforming a commercial T-tau assay, which failed to differentiate between CU and AD samples in our cohort, the P.pAb-P.pAb assay appears to provide a better approach for measuring tau in plasma. While these findings suggest an improved ability to capture disease-relevant changes, further validation against CSF tau fragments and tau PET imaging will be important to confirm whether this assay more directly reflects underlying AD pathology.

## Supplementary Information

Below is the link to the electronic supplementary material.

Supplementary Material 1

## Data Availability

All data are provided within the manuscript or the Supplementary figures/tables. Data and materials are available from corresponding author, Mohammad Arastoo, on reasonable request.

## Abbreviations

2N4R tau: Full-length tau isoform (1-441 AA)
aa: Amino acid
AD: Alzheimer’s disease
CI: Confidence interval
CSF: Cerebrospinal fluid
CU: Cognitively unimpaired
dGA: Tau297-390 derived from 2N4R tau isoform
ELISA: Enzyme-linked immunosorbent assay
EM: Electron microscopy
FTD: Frontotemporal dementia
HRP: Horse radish peroxidase
IgG: Immunoglobulin G
IP-MS: Immunoprecipitation-mass spectrometry
kDa: Kilodalton
LLOQ: Lower limit of quantitation
mAb: Monoclonal antibody
MTBR: Microtubule-binding region
NINCDS-ADRDA: National Institute of Neurological and Communicative Disorders and Stroke-Alzheimer’s Disease and Related Disorders Association
NfL: Neurofilament light
nM: Nanomolar
PBS: Phosphate-buffered saline
PBST: Phosphate-buffered saline plus Tween® 20 (0.1%)
PET: Positron emission tomography
pAb: Polyclonal antibody
P.pAb: Proline polyclonal antibody
P.pAb-P.pAb: Proline polyclonal capture, proline polyclonal detection assay
Pm: Picomolar
pTau: Phosphorylated tau
pTau217-P.pAb: PTau217 capture, proline polyclonal detection assay
pTau217-Tau12: AlzPath commercial Simao pTau217 kit
ROC: Receiver operating characteristic
RGP: Resorufin β-D-galactopyranoside
SD: Standard deviation
SDS-PAGE: Sodium dodecyl-sulfate polyacrylamide gel electrophoresis
SBG: Streptavidin β-galactosidase
Simoa: Single molecular array
SPR: Surface plasmon resonance
TEM: Transmission electronmicroscopy
TMB: 3,3′,5,5′-Tetramethylbenzidine
T-tau: Total-tau
