# Supplementary material for: Quantification of plasma tau species containing the proline-rich region as a biomarker in Alzheimer’s disease
Source: Sci Rep. 2025 Nov 25;15:41881. doi: 10.1038/s41598-025-25864-x (PMC12647631; doi:10.1038/s41598-025-25864-x)
Supplement: Supplementary file 1 — Supplementary Material 1 [file 41598_2025_25864_MOESM1_ESM.docx]

**Supplementary Figures**


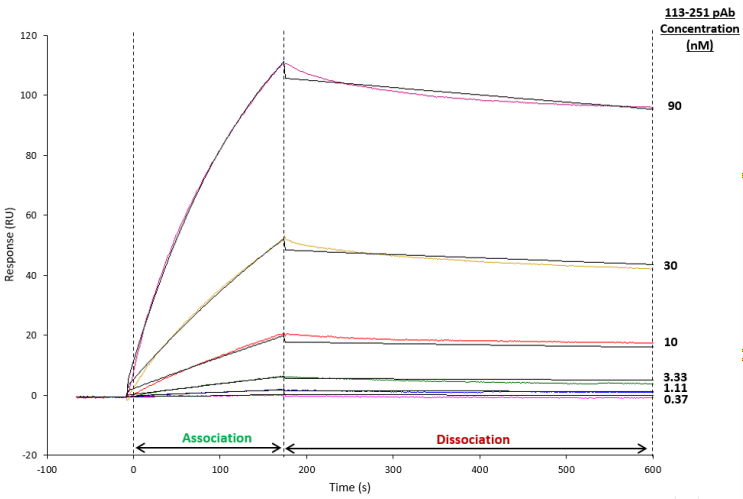


**Figure S1**: Multi-cycle kinetics of P.pAb binding to 2N4R tau. Increasing concentrations of pAb

(0.37 – 90 nM) were passed over a CM5 chip immobilised with 200 RU of amine-coupled 2N4R tau for 180 s. The chip was regenerated between each cycle following a 420 s dissociation phase. Data fitted to a 1:1 binding model using Biacore X100 evaluation software. Coloured lines = raw data, black overlaid lines = computer-modelled fit. pAb KD was calculated as 3.5 nM.


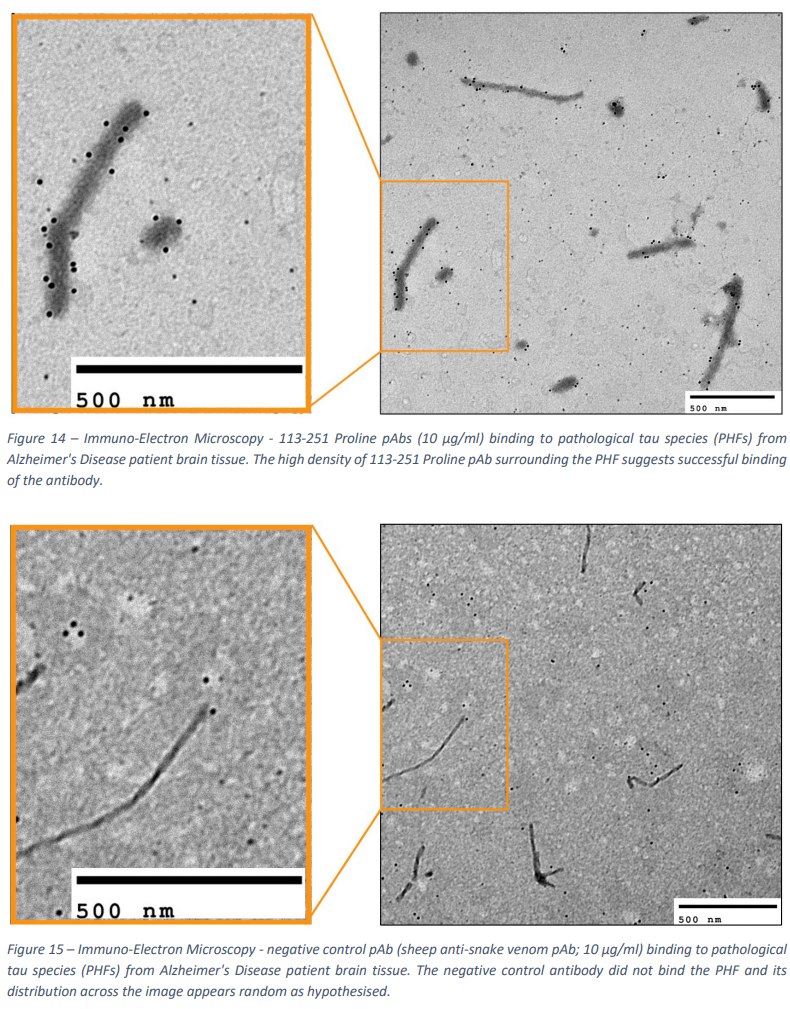

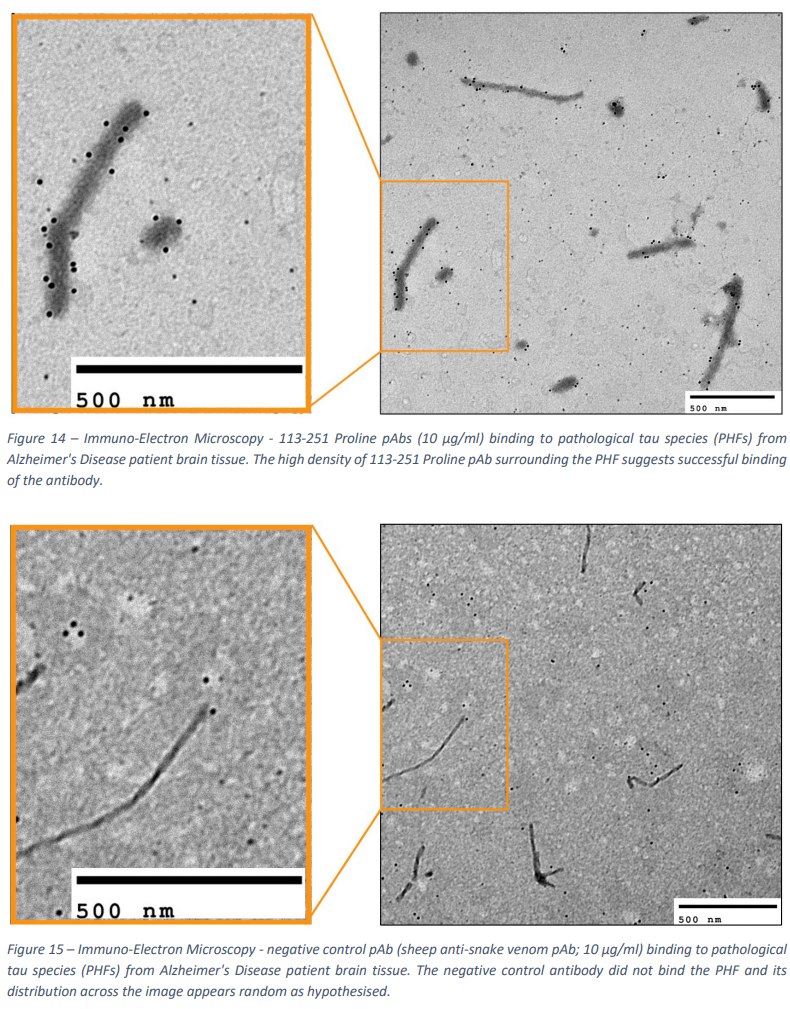

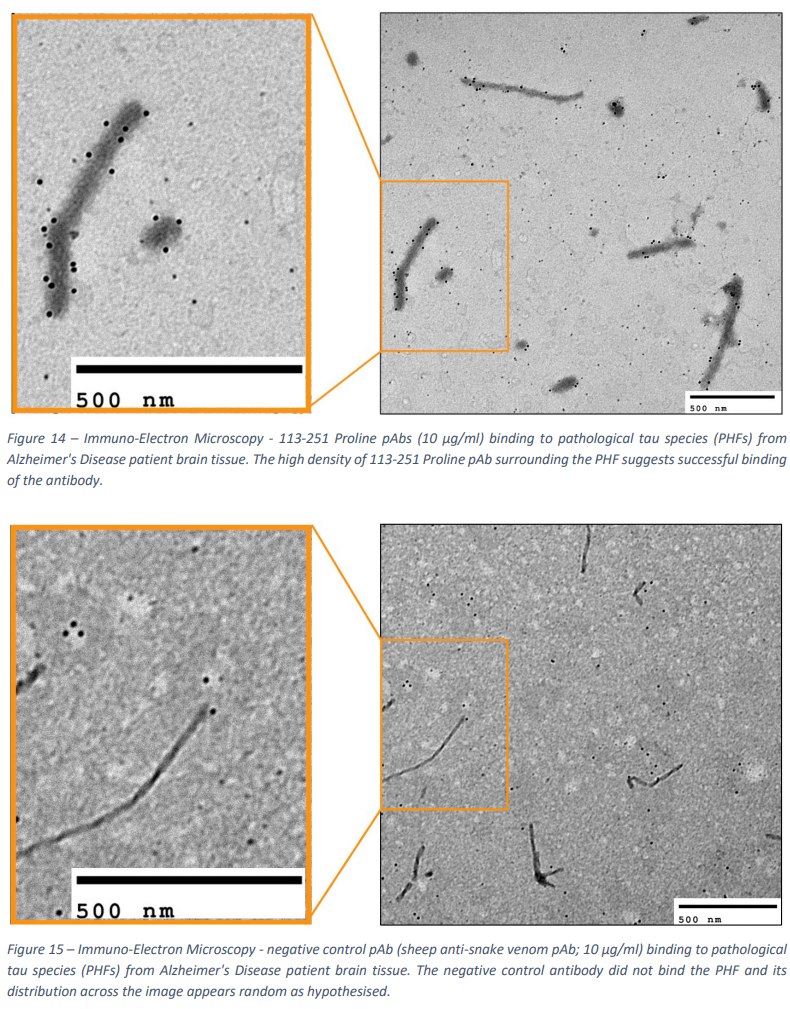

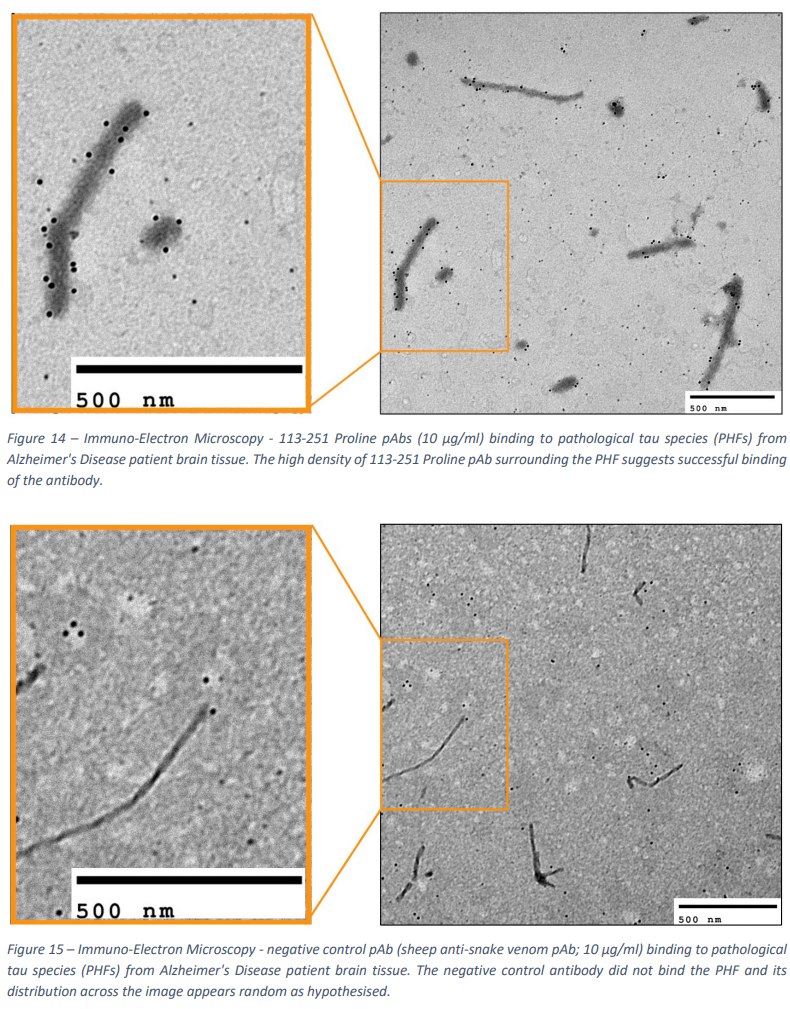


**A**

**B**

**Figure S2:** Immunogold staining of tau filaments derived from human AD patient frontal cortex A) P.pAb used as primary antibody resulted in binding to all PHFs on the EM grid. B) Negative control pAb used as primary antibody did not result in any binding. Primary pAbs were used at a concentration of 10 ug/ml and 10 nm gold-conjugated anti-sheep IgG used as secondary antibody. Images taken at X 50,000 magnification using TEM JEOL 1400 plus. Scale bar = 500 nm. An example PHF has been enlarged to provide a more detailed view for each condition, as indicated by the black box.

**
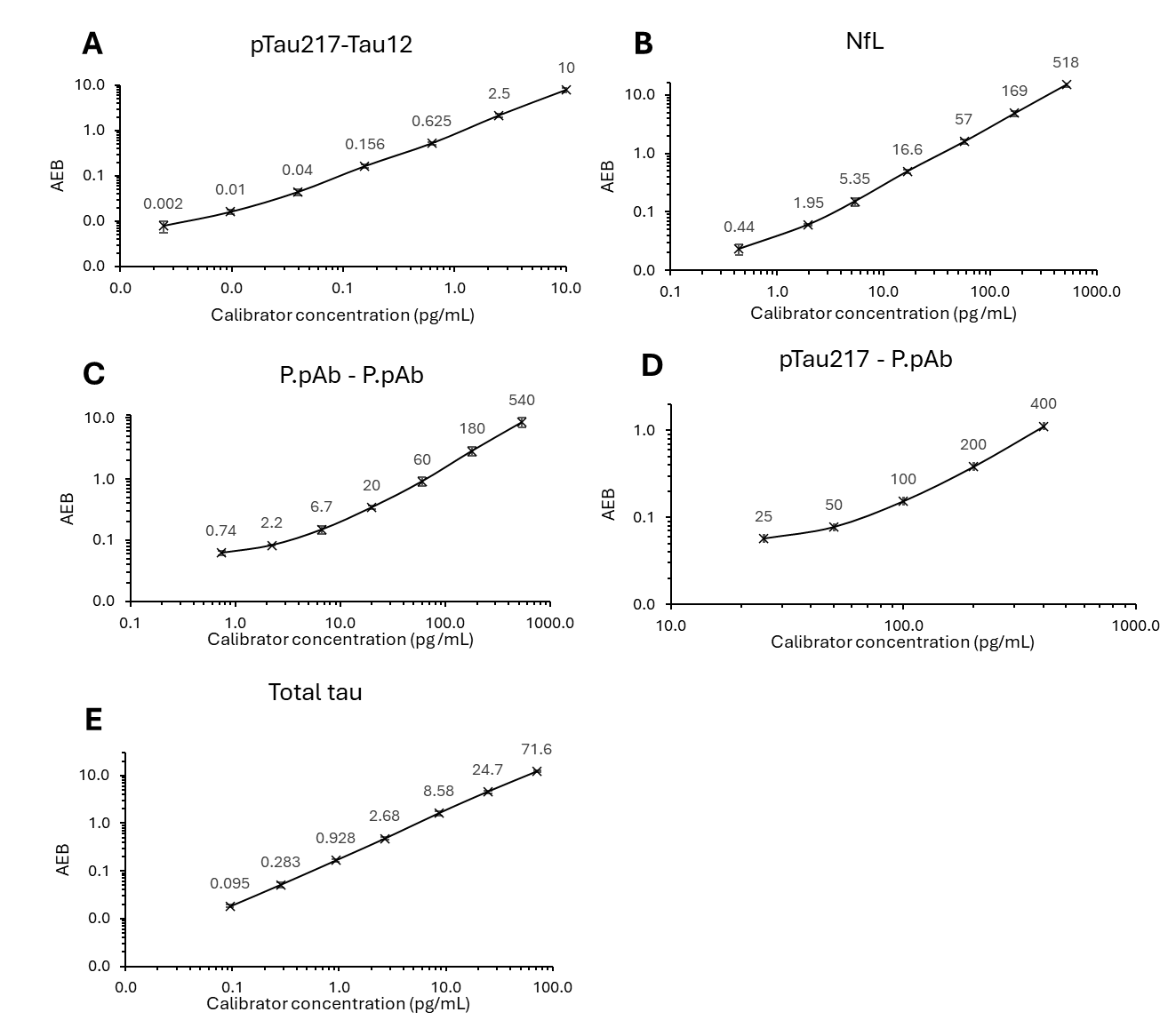
Figure S3**: Standard curves for various assays on which TRx-GTD-025 samples were assessed. Where stated, the first antibody denotes the capture, and the second antibody denotes the detector. Error bars represent standard deviations from 3 repeats of the standard curve on different days.

**
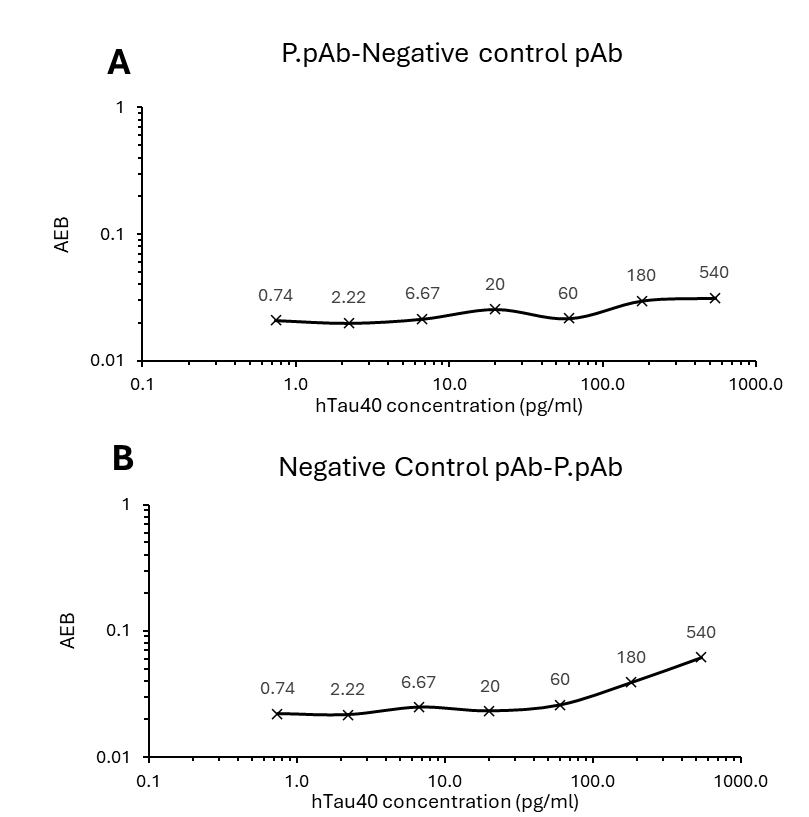
**

**Figure S4:** Standard curves for A) P.pAb capture paired with negative control pAb detector and B) negative control pAb capture paired with P.pAb detector in Simoa experiments, following the same methodology as the P.pAb-P.pAb assay.

P.pAb-P.pAb assay validation

Lower limits of quantification were defined by running a blank sample (tau 2.0 diluent) in triplicate and adding three standard deviations to the measured mean value. This was determined to be 2.2 pg/ml. The upper limit of quantification (ULOQ) was set as the concentration of the highest calibration point which was 540 pg/mL. To assess parallelism, a pooled plasma sample (containing K2 EDTA anticoagulant, BioIVT) was assessed with a 2-fold dilution series (1:4, 1:8, 1:16 and 1:32) (Fig. S5 A). For spike recovery, the pooled plasma sample was diluted 2-fold (1:4, 1:8, 1:16 and 1:32) and spiked with 20 pg/mL 2N4R tau (Figure S5 B). Percentage recovery was determined based on the equation: % Recovery = concentration of spiked sample ÷ (concentration of non-spiked sample + concentration of spiked buffer). Recovery was 108 %, 101 %, 85 % and 96 % in plasma diluted 1:4, 1:8,1:16, and 1:32 respectively. To assess selectivity, the pooled plasma sample was diluted 1:4 then spiked with 20 pg/mL dGA (-ve ctrl) or phosphorylated 2N4R tau (ph.2N4R, performed in duplicate). While quantification increased by 0.8 pg/mL in the dGA spiked sample, it increased by 14.2 pg/mL in the ph.2N4R tau group (Fig. S5 C). Furthermore, a sample of 0.5 ng/mL ph.2N4R tau in diluent returned values above the ULOQ to a quantification of 0.75 ng/mL, whereas a sample of 0.5 ng/ml dGA in diluent returned signals below blank (data not shown). A further validation check was assessment of plasma pooled from 3 x tau KO mice (The Jackson Laboratory, USA) and 3 x L66^+/+^ transgenic mice, which overexpress human tau protein. While signals for tau KO mice were below the LLOQ of the assay, 42.1 pg/mL was quantified in the pooled L66 mouse plasma sample (Fig. S5 D). Precision and accuracy were tested by measuring two replicates of two control plasma samples, with low and high P.pAb-P.pAb quantification, on two consecutive days. Average intra- and inter- assay %CV were 7.15% and 13.8%, respectively (Fig. S5 E). Furthermore, to ascertain signals are not being derived non-specifically, from the P.pAb capture or detector alone, P.pAb was paired with a -ve control pAb for capture and detection. These resulted in no standard curve or detection in any sample that was assessed (data not shown).

**A**

**B**

**C**

**D**

**E**

**Figure S5**: Validation parameters assessed for P.pAb-P.pAb assay. A) Parallelism was assessed in a pooled plasma sample diluted 1:4 following a 2-fold dilution series. B) Spike recovery was assessed in a pooled plasma sample diluted 4, 8, 16 and 32-fold and spiked with 20 pg/ml 2N4R tau C) Selectivity was assessed by spiking a plasma sample with dGA (-ve ctrl tau aa 297-390) and phosphorylated 2N4R tau. D) Selectivity was further assessed by quantification in plasma from transgenic tau KO mice and L66 transgenic mice. E) Two control plasma samples were utilised to assess intra- and inter- assay coefficient of variance. FC, Fitted Concentration.

pTau217-P.pAb assay validation

LLOQ and ULOQ were determined to be 25 pg/ml and 400 pg/ml based on parameters mentioned above. Precision and accuracy were assessed by measuring two replicates of control samples, with intra- and inter- assay % CV of 2.8 % and 2.6 %, respectively (Fig. S6 A and S6 B). Assessment of transgenic mice showed signals below blank in a pooled Tau KO plasma sample diluted 1:4 (The Jackson Laboratory, USA), whereas 872.2 pg/ml was measured in a pooled L66^+/+^ mouse plasma sample (Data not shown). Due to the low intra- and inter-assay % CV of this assay, it was deemed suitable to run samples in singlicate.

| **Sample**  **A** | **FC (pg/ml)** | **Avg. FC (pg/ml)** | **Intra-assay % CV** |  | **Sample**  **B** | **FC**  **(pg/ml)** | **Avg. FC (pg/ml)** | **Inter-assay %CV** |
| --- | --- | --- | --- | --- | --- | --- | --- | --- |
| Ctrl 3 replicate 1 | 817.5 | 813.9 | 0.6 |  | Ctrl 5 replicate 1 | 245.6 | 248.9 | 1.9 |
| Ctrl 3 replicate 2 | 810.3 |  |  |  | Ctrl 5 replicate 2 | 252.2 |  |  |
| Ctrl 4 replicate 1 | 1179.2 | 1138.6 | 5.0 |  | Ctrl 6 replicate 1 | 643 | 658.3 | 3.3 |
| Ctrl 4 replicate 2 | 1098 |  |  |  | Ctrl 6 replicate 2 | 673.6 |  |  |

**Figure S6**: Control plasma samples were assessed for (A) intra- assay %CV by analysing samples in duplicate on the same plate and (B) inter- assay %CV by analysing samples in singlicate on different plates and different days. FC, Fitted Concentration.

**Supplementary Tables**

| **Antibody** | 2N4R tau EC_50_ (nM) | 113-251 tau EC_50_ (nM) |
| --- | --- | --- |
| P.pAb | 0.14 | 0.087 |
| CC7 mAb | 1.01 | 0.55 |

**Table S1**: Summary of EC50 values of P.pAb and in-house generated CC7 mAb (Tau145-157) against 2N4R tau and 113-251 tau peptide. EC50 (nM) was calculated from binding curves generated by ELISA with double dilution of antibody at 13.33 nM across a 96-well plate coated with respective antigens. In comparison to CC7, P.pAb showed 7.2 fold improvement in binding to 2N4R tau and 6.3 fold improvement in binding to 113-251 peptide.

| **Biomarker** | **AUC (95 % CI)** | **Improvement in AUC** | **Improvement in sensitivity (%)** | **Improvement in specificity (%)** |
| --- | --- | --- | --- | --- |
| P.pAb - P.pAb | 0.808 (0.696-0.920) | 0.077 | 12 | 0 |
| pTau217 - P.pAb | 0.904 (0.8280-0.980) | 0.144 | 27 | 10 |
| pTau217 - Tau12 | 1.0 (1.0- 1.0) | 0.161 | 19 | 23 |
| NfL | 0.864 (0.773-0.955) | 0.039 | 12 | 0 |

**Table S2**: Improvements in AUC, sensitivity and specificity of assays following removal of AD samples with low pTau217 concentrations (below cut-off point determined by Ashton et al. 2024) from selected cohort.
